# Micropsychology

**Published:** 1862-01

**Authors:** 


### Art. IX.—MICROPSYCHOLOGY.

This is an age of big words. Everyone who has bled or blistered or nauseated a maniac, or who, more pretentiously, is entrusted with the superintendence of a ward of lunatics, is a Psychologist. But in a large majority of cases the possessor of this proud and pompous title knows nothing of the human mind with which he professes to deal; of that subtle structure which he proposes to re-establish; of those intellectual and moral laws which it is his duty to regulate. But even where no such ignorance exists, where the alienist can repeat the alphabet of his science glibly, there is often a total blindness or apathy or indifference to the vastness and importance and the real characteristics of the sphere in which every physician intrusted with the care of the insane daily and hourly moves. There are observers, starting from the Dan of idiocy, and journeying on and on till they arrive at the Beersheba of moral insanity, who declare all to be barren. We have traversed galleries containing hundreds of patients, and have been assured that among them there was no case of interest. There was profound philosophy in the nursery tale of "Eyes and no Eyes," for there are minds, and powerful although not acute minds, so constituted that they fail to detect not merely the "good in everything," but matter for instruction and investigation in the barrenness, in the harmony or disorder or sameness, in the soil upon or out of which these grow, or the substratum which that soil covers, or in the footprints on the sand, or the rain-drops which have effaced them. It is not rare or marvellous or striking cases alone which convey knowledge or command interest. These fall legitimately to the curious, the superstitious, the miracle-mongers in science; who disregard a truth because it is small and ordinary, and prize and record an observation because it is unique. These errors have, perhaps, exercised an influence over every mind; we have all been too much disposed to take comprehensive views of mental diseases, we have been embarrassed and distracted by the wideness of the field in which we are called to labour, by the weight of the responsibilities by which we are surrounded; we have been engrossed by the grand outlines, the distant horizon, the grave difficulties and obstructions, and the rapid movement in our course, and have lost sight of minor and nearer objects, and of their significance and bearing upon every advance and arrangement which may be attempted. We have exhausted our energies in struggling with mania, in defeating suicide, in soothing suffering, in supporting the dying; and the ministrations are noble; but we have passed unheeded the circular walk, the sibillant cry, the single spot of anæsthesia which a demon has touched. There is not much time in the hurry of practice to study such peculiarities, such minute points, especially as medical treatment is applied to the culmination or crisis of disease rather than to its course; yet every feature and act in the insane should be accepted of as a sign or symptom or premonition, or both, the value of which can only be determined by investigation. A philosopher gained a world-wide reputation by observing that a line was the result of the meeting of two different colours; and a physician claims credit that the tremor of the upper lip or tongue has been accepted as a fatal prognostic in one form of derangement, while the tone of the voice is supposed to herald sanity and serenity in another. The microscope is now universally and so constantly employed in determining the origin, the nature, and the treatment of many physical diseases, as to suggest the application of more minute observation to the phenomena of insanity; the collection and appreciation of symptoms which have been hitherto disregarded, or designated harmless extravagances and idiosyncracies which it was safe to ignore; or vicious and troublesome habits and practices which it might be incumbent to combat, but which it would be idle and unnecessary to understand or describe. Since the days of old Arnold, little attention has been paid to this subject, and even he confined his illustrations chiefly to hypochondriacal insanity. But there are numerous indications which cannot be included under the categories alluded to above, nor are entitled to be identified with what our neighbours call "*tics*," and which, from their apparently trivial or transitory or exceptional nature, have not been noticed at all, or noticed very cursorily; but which must have a meaning, and relations, and influence, and must less or more, when understood, enlighten the judgment as to the mental condition of the patient in whom they are observed, whether such enlightenment may modify the principles of treatment or not. It is certain that every act of the insane, however absurd, inexplicable, inconsistent with former character or existing pretensions, proceeds from a motive; and is willed in order to accomplish a certain object. It is unphilosophical, often cruel, and pregnant with serious consequences, to infer from the act, in accordance with healthy and recognised rules of conduct, the propensity or conviction, or the lack of these, from which it proceeds, and to award reprobation or praise, and to consign the actor to a particular class and position, as if in possession of the whole truth. There is undoubtedly a philosophy of the insane, as well as of the sane mind; there are laws or modes of action which supplant or succeed those which have been enfeebled or eradicated; and which can only be understood or conjectured when the whole of the phenomena, when the subjective as well as the objective elements, are embraced. Esquirol had under his charge a patient who daubed the walls of his room with fæces. Every night was devoted to tracing wild and fantastic figures upon the pure and elegant paper. He was stigmatized as of degraded tendencies; his debasement was pronounced a proof of dementia, and he was doubtless subjected to the stern and repulsive management which, even under the benign rule of his physician, awaited such a doom. He recovered; and then revealed that he had been an artist; that he had been deeply impressed by the paintings in the Louvre; that he regarded his excrement as gamboge, and that, in the absence of all other materials, he was constrained to preserve the glorious imaginings with which he was favoured on the walls of his cell. It is highly probable that in seeking out and selecting the infinitesimal or partially observed phenomena which it is desirable should assume an acknowledged place in diagnosis, the same course must be pursued as in the case quoted above, and it may be either impossible to assign any elucidation of the fact recorded, or it may be necessary to adopt an elucidation purely conjectural. The inquiry may be pursued in several ways. The analysis may be carried into the minds of the sane, and even of those who are the types, the giants of their time, the polished corners of the temple: and the oddities and inconsistencies, the hidden but hideous appetites, or the puerilities and littlenesses which bring them close to the verge of insanity, destroy and desecrate the sanctity and loftiness of the hero or martyr or philosopher, and affect the trustworthiness or sincerity or comprehensiveness of their deliverances, may be brought to light; or, embracing the whole range of mental disease, but omitting the palpable and recognised symptoms, the attention may be directed exclusively to modes of thought or speech or action which are less obtrusive, which require to be sought for, which may be carefully concealed, which may be unknown to the patient and those around, or if known, may be viewed altogether apart from the malady under which he labours. Or faint traces of morbid thought may be sought for among the constitutional changes which attend the processes by which the frame is nourished, modified, degenerated, destroyed; or even among the crimes and enormities which remove human nature so far beyond our ordinary sympathies as in one sense to isolate the perpetrators as a distinct race of beings.

In inaugurating a new aspect of an old subject, it may be best to avoid rigid adherence to either of these modes of investigation, but to pursue all, and to appropriate materials from the subjects to which they are directed, so far as may be subservient to the object proposed. Were the proposition advanced categorically, that a certain contour, not the physiognomy, of the countenance was associated with idiotic and weak mind, it would be received with incredulity, or the coincidence would be referred to that general deformity which characterizes this class of beings. But this is true, not only as a general law, but there exists a strong probability that different forms of face distinguish separate classes of idiots; that there is the round-visaged idiot, the general contour of whose features and whose eyes and mouth are circular, who is dwarfish, obese, and rotund; and the oblong-visaged idiot, whose features are elongated, gibbous, and acute, and whose frame is gracile and attenuated. The teeth decay prematurely in these individuals; they are diminutive or disproportioned in size, and irregularly set. There is often present the cleft lip of the scrofulous child; and, what is more marked and permanent, although until lately it escaped observation, the cleft, the vaulted, the carinated, or the conical palate. Such features acquire their true importance and their relation to psychology, when viewed as parts and proofs of that malformation or arrest of development which involves or influences the nervous system, and upon which congenital idiocy so often depends. And were the argument pushed further, and the assertion made that not merely the xanthous and melanous tribes of the human race, but the beautiful and ugly members of these natural divisions, are exposed to insanity, and, there is a probability, to different kinds of insanity in different proportions, the statement would be derided and denounced as an attempt to carry out the law of uniformity of sequence to an unwarrantable extent. Yet statistics justify this and still more minute distinctions, and appear to demonstrate that individuals with chestnut or brown are more prone to insanity than those with dark eyes as 102 to 17; that the tall are more exposed than the short, as 102 to 19; and that the moderately spare are more exposed than the fat, as 122 is to 6. These must not be viewed as isolated conditions, but as signs of temperaments and diatheses which are conjoined with certain invariable tendencies and susceptibilities of the nervous system.

There are certain lunatics who always greet with the left hand, or with a finger of that hand. This mode of salutation may be determined by the desire to conceal real or imaginary paralysis, by the unworthiness of the person addressed, or because the right hand is blood-stained or criminal. They may touch, or rub, particular parts of their own body, or of that of their companion, and we have recently seen a patient who moved one hand rapidly around the other. When the law of hereditary transmission is traced in the inheritance of striking mental or physical qualities, or of mental defects and diseases, or of tendencies to crime, the phenomenon is recognised as natural, though marvellous; but it is found that the taint may comprehend all and the most insignificant peculiarities, and may extend even to such trivial acts as those alluded to. A distinguished Professor has related to us the following anecdotes:—A nobleman of shy and retired habits, slow of utterance, and awkward in manners, invariably rubbed his hands long and energetically and silently, when overjoyed or powerfully affected, and as a preliminary to articulate expression. He did so when the birth of a son and heir was announced to him. He died when his successor was still an infant. The child when about ten years old was most anxious to obtain a miniature working steam-engine. His mother and friends pertinaciously refused the gratification of his wish. The narrator was, however, permitted to give the toy, and was much struck to observe on presenting it that the child did not speak his gratification, but rubbed his hands long and rapidly, precisely as his father had done, whom he had never seen.

A man of highly nervous and morbid disposition and solitary life, was observed to avoid the contact of metal, especially the handle of a door. If he opened the door he protected his hand with his coat, or in some other way. His son, who scarcely ever associated with him, and only in his own house where the doors were left ajar, or opened by others, on his first attempt, and ever subsequently, covered his hand before he touched the lock. We have seen the secretion and hoarding of small and useless articles in places where they could neither be preserved nor recovered, in two generations of excitable temperament; we have seen the reproduction of the same grimaces and distortion of countenance in an insane niece who never met an eccentric uncle who grinned and squinted in the same way; and we have known a daughter and a mother both believe that they laboured under imperfect vision. But a celebrated historical example of hereditary delusions is afforded in the family of the regicide Oxford, whose father and grandfather, as well as himself, declared that they were St. Paul.

It is well known that particular phrases and ridiculous modes of articulation reappear in different insane members of the same family, either from imitation, or as an expression of the same predominating dispositions or delusions. The phenomena of the voice, articulation and language, afford more ample opportunity for the detection of those slight but important departures from health, which it is the present object to record, than any other class of psychical acts. These manifestations are sometimes the consequence of physical changes, such as in paralysis; sometimes they are the acts of a perverted will; and they are sometimes the expression of hallucination, as when the divinity or demon that dwells within constrains the patient to utter that which is repugnant to his own nature and what he knows to be inconsistent with truth.

There is met with in mania and theomania extreme volubility: an irresistible rapidity and copiousness of utterance, which although overlaid by more important symptoms, is characteristic of the exalted passions and enfeebled self-control which exist in these affections. There is likewise the involuntary use of certain words and phrases: a repetition which does not proceed from the acceleration of thought or from the inability to select and articulate other words. Cases are narrated, especially in French authors; and when a student in Salpetrière we long watched two illustrations of this defect. In one the only word used was "Adeline," in the other at all times and under all circumstances, and to the exclusion of every other expression, the sufferer repeated "*Je suis immortelle.*" In a case under our own care a theomaniac uttered constantly, in defiance of commands, threats, under the penal operation of the douche, and when his head and face were covered with stucco during the process of taking a cast, "Bless the Lord, bless the Lord's God, bless the Psalm Book," and on opening the said book, which he carried reverently in his bosom, he selected and read, "Bless the Lord, oh my soul." Romberg mentions repetition as an indication in cerebral softening; but it is the repetition of words addressed to the patient. He calls it the "echo sign."

In Charenton there was, many years ago, a case under observation in which all classes of words were obliterated from the memory except substantives. This forgetfulness is occasionally confined to nouns, while verbs are preserved, and it is believed that cases occur in which every part of speech in turn has been lost; but in P. L. a copious vocabulary furnished him with no reply to a question as to his health, save "honneur," "courage," "espérance," "Dieu," which was, circumlocutively, interpreted to mean "that he was honoured by the inquiry, that he was of good courage, and trusted in God." This oblivion is sometimes confined to particular words without any reference to their meaning or grammatical relations. An individual in the ordinary course of conversation, or in the impetuous rush of a declamatory harangue, stops suddenly; a phrase is wanting, which no exertion nor consideration can recall, and which never is recalled. A patient's own name has been blotted out. Parts of words, and always the same parts, are lost sight of in general paralysis. At an early stage of the discovery of this malady, it was observed by Calmiel and others that the last and penultimate syllables were blurred, or imperfectly and slovenly pronounced, "entrecoupées," in this affection; and the deficiency was attributed to paralysis of the tongue; but subsequently the same omission was detected in the writings of such patients; and the observation suggested the belief that the infirmity was psychical or psycho-physical, rather than muscular. The laws of orthography are utterly disregarded even by educated men when affected with this disease; but an example of this and of the most microscopical mental affection in relation to oral language that can well be conceived, where a single letter was alone remembered of each word, may be selected from another form of disease.

A farmer, who had been affected with paralysis, appeared to retain his memory for all parts of speech except nouns substantive and proper names; the latter he could not at all retain, [illegible] this defect was accompanied by the singular peculiarity that [illegible] perfectly recollected the initial of every substantive for which he had occasion in conversation, though he could not recall to his memory the word itself. Experience, therefore, taught him the utility of having written a list of the things he was in the habit of calling or speaking, including the proper names of his children, servants, and acquaintances; all these were arranged alphabetically, in a little pocket dictionary, which was used as follows: if he wished to ask anything about a "cow," before he commenced the sentence he turned to the letter "C," looked out the word "cow," and kept his finger and eye fixed on the word until he finished his sentence; if about to go to "Dublin" he turned to "D," and so on.*

* Graves, *Dublin Quarterly Journal of Medical Science.*

There may exist limitation to particular words where, with the capability of articulating others, the individual is restricted to "snuff," "papa." Hoffbäuer cites cases in which words were habitually transposed, as "rose beautiful is." Dr. Shapter has given the case of Dr. P. Gillio, who, after an attack of cerebral excitement, transposed or arranged his words irregularly and stenographically, in writing; and the experience of every physician may recall instances of the substitution of one phrase or sentence, for another. In the first stage of excitement, and sometimes in chronic mania, a jargon is resorted to either by coalescing and confounding words, or as an expression of passion; and in affections of which fanaticism is an element these uncouth sounds assume a more systematic arrangement and vaticinatory import, and are proclaimed to be unknown tongues, the direct inspiration of Deity. We have known the voice permanently lose an octave in chronic recurrent mania; Morel has noticed that its sharpness and shrillness announced an attack of insanity. It may become uncontrollable, and be prolonged into a howl or a shriek in imitation of the lower animals, and perhaps in accordance with their propensities and instincts. The cry of the carnivora and the song of birds may often be heard where human language is ignored and avoided. Natural signs have been resorted to in preference, and in place of sounds; and where such have been necessarily adopted, atomic deviations from health and accuracy have been detected in them. A deaf mute laboured under general paralysis, and towards the close of life he not only wrote his delusion, that he could speak, in the imperfect and incomplete manner paralytics do, omitting the terminations of words; but he spoke incoherently on his fingers, and lost the knowledge of the digital alphabet gradually, recollecting a few of the signs, such as S and H much longer than others, and repeating them incessantly in his vain endeavours to render himself intelligible. This singular case goes far to demonstrate that the symptom illustrated is exclusively of mental origin. Guislain detected a difficulty in articulating T and R in paralytics. The mutism frequently characteristic of melancholia and an obstinate disposition is voluntary, and may be dissipated by sudden emotion, or a judicious application of the shower-bath. Vanity may lead to the mispronunciation of words, but the use of diminutive and infantile expressions cannot be explained upon this ground; and the grotesque and extravagant modifications introduced suggest another origin. A patient for three consecutive days vociferated incessantly words terminating in *ation*, or rather he added to every word that occurred to him that syllable. One class of patients sing or chant whatever they have to say; another rhyme their incoherence. The versified ravings of an aged chronic maniac were taken down. One improvisation commences—

> Jemmy Edgar was a joiner
> But never a penny-a-liner;
> He delved, but never span,
> A regular gentle gentleman.
> He crossed the Equator in a ship,
> With a jump and a skip.
> If he fell, deil may care,
> There's better men in Stranraer.

M. Billod has given a specimen of incoherence in verse. The lines were not, however, extemporized, nor did rhyme form the patient's ordinary mode of communication. The composition is part of a poem produced by a patient labouring under what is styled "geographic or historic association," in which places and persons seem to suggest the course of the thoughts:—

> Viens, viens, mon très cher Eugène,
> Viens, viens, revoir ta carène,
> L'Indoste suit toujours Tamerlan;
> Tu prends le casque de l'eperlan;
> Tu vas renaître sur le mont Acide
> On y place l'etendard d'Acide.
> Tu porteras chez nous la sainte dague,
> Tu verras les clochers de Copenhague.*

Incoherence in itself affords many faint shadowings of the disruption of recondite mental relations, of errors within errors; but quotations showing the effect of association of ideas directed by the sound of the sign, must suffice. An illustration of a modification of this relation was afforded in the case of a person, who could still construct his sentences according to the ordinary mode, but who was guided in his choice of expressions by the sound of the terminal syllable or word, or by some rude notion of rhyme. So dominant and necessary did this tendency appear to be, that he paused to consider or discover the appropriate word; sacrificed every pretension to sense or reason, and embraced every incongruity and absurdity with a view to accomplish his object. If he concluded a phrase by the word "remorse," it was certain that horse or worse would occupy a similar place in that which followed; if he used "firkin," gherkin was immediately suggested; and while he continued to make his harangue a vehicle for his wishes, and for sneers at those around, if he failed to summon up a term which harmonized with "coverlet," he

* "De la Lésion de l'Association des Idées," p. 550. *Ann. Med. Psych.*, Oct. 1861.

immediately adopted *plover*—wit, or some term equally euphonious and absurd.

In a female, whose disease was, in other respects, different, an analogous peculiarity of language existed. Her mind was likewise directed by the sound, but there was no attempt to use verse as a model. Her choice was generally determined by the initial letters of the principal word of the paragraph, so that her conversation assumed an alliterative form. Some of her observations follow—"The stick she had was the handle of a pick to dig potatoes, and peas, and plums; but the dog dragged the dust through the mignionette, and made sad work with the willow wands, and the sands on the sea-shore. Give me that book, the crook of the blot—Lot's wife was a witch, and a pillar of salt and of sorrow." A lady, transferred from a less splendid residence, concluded that she had arrived at the palace of a nobleman, that she was attended by his menials, supplied with viands from his table; but through a long series of delusions preserved a natural and congruous sequence, each successive irrational supposition being the legitimate suggestion of the antecedent. It was further remarked that this singular exemplification of the law of simple suggestion could, almost at all times, be observed in her language. Amid great incoherence it was evident that a word in one sentence almost invariably suggested the succeeding thought and sentence. The following paragraph may be said to have been dictated by her. "The shells, the beautiful pink *shells* cast upon the sea-shore, require fifty days to consolidate; but then *marble coffins* are expensive, and will not make into *statues*. And then they speak of their *Venus*, but for my part I would rather go to Carlisle than *Venice*, for I have an old *pier* glass that my ancestors got from Lord *Stair*, and he was a *peer*, and he had *steps* to his castle, and did not *wander* from our communion, for it takes *fifty* years for their progress. Mr. S. was a *hundred* and *four;* but what is *time* to the fair flowers, and the *thyme* that feeds the birds and the bees."

A gentleman came under our notice the cause of whose derangement was centered in a Greek particle, and many patients conceive themselves to be polylinguists and fearlessly proffer proofs of their proficiency. Soliloquizing and somniloquizing are the most common, but little heeded indications of morbid affections of the faculty of language. Individuals write only in Greek, or in old Saxon characters; they write from right to left, or perpendicularly; they occupy the whole of the paper to the remotest corner and most linear margin, whatever the size of the sheet or the amount supplied may be; they commence all their words with capitals, or introduce them in the middle: they devise characters; they repeat for years and without limit the lineaments of human faces arranged in rows, and in one case the occupation during the whole of the attack was the rude delineation of thousands of human skeletons.

Discipline has caused the disappearance of many of these petty peculiarities. The insane, though as unhealthy, are now drilled and trained into the semblance of sanity and order; they are cast very much in the same mould; and it is a tribute to the efficacy of modern management to say that they have lost much of the piquant absurdity and picturesqueness of former times. Yet in any given group of lunatics, these shadows, "nuances," may be noted. Not only is the hesitating speech the centre of a history of vain ambition and imaginary aggrandizement, but the posture may reveal a man's downward destiny, and the position of the thumb may point to serious alterations in the membranes of the brain. Attitudes and progression in the insane may be first the natural language of the delusion; or, secondly, they may be symptoms of the condition upon which the delusions depend; or, thirdly, they may accompany morbid states of the instinctive parts of our nature. A patient, who, under the impression that the solid and substantial wall, or the world itself, is tottering to its fall, exerts all his strength and presses energetically against the fabric in order to prevent the catastrophe, is an illustration of the first proposition; another, who cannot preserve his equilibrium from vertigo, is an example of the second; and he who leaps, vaults, runs, wrestles without assignable motive, must be placed under the third category. These affections are differently influenced by volition. They may be the direct dictate of the predominating idea, or emotion, consciously and deliberately and pertinaciously expressed, as when an individual burrows the head between the knees to prevent the hideousness of his countenance from being seen. They may be acquired instinctively during abstraction, without the consent or co-operation of the will, as when a melancholic twirls an imaginary thread for months between his fingers. They may exist in opposition to and uncontrollable by the will, as in hysterical movements, or where the aid of others is claimed to prevent acts of violence, or gesticulation. And they appear altogether independently of volition, as a consequence of its abolition, as in the various modifications of convulsion.

There are classes of patients who touch, pick, abrade a particular spot either in their own body, or in the surrounding objects. This may indicate an uneasy sensation, or the existence of a delusion associated with the part, or object touched. An individual carried his hand to his head avowedly because he experienced discomfort there, and believed that his brain was either abstracted, or converted into stone; another made himself bald by irritating an ulcer of the scalp ; another denuded the face to remove a sensation of creeping; two produced sores on the epigastrium as a means of allaying deep-seated pain. There is in every asylum a group of inmates who may be called attitudinizers, who would, if undisturbed, indulge in the same position for months and years, although they may not display any preference either for the same place or for places of the same kind. They may be seen prone, or supine, upon the floor, or standing stiff and rigid as a pillar; or crouched and huddled up furtively in corners, or upon chairs or sofas. The incentive to the adoption and preservation of such positions may be apprehension, associated with painful sensations in the abdominal viscera; or a sense of shame or dignity, or merely the craving for that muscular relaxation and perfect repose which to the enfeebled mind amount to enjoyment. There are others who invariably occupy the same spot, and whose movements may be said to depend upon the will of others, and of whom it was truly written, Sedebit solitarius et tacebit. Again, we occasionally meet with those who not only select a particular spot, but grovel and burrow in it, where that may be practicable ; and who crawl and contract themselves into the smallest possible space under benches or near the fire-place. Many of these patients creep, or move, serpent-like, and in one case we have seen the movements entirely quadrupedal, but performed in repeated and rapid bounds like the leaps of the marsupial animals, notwithstanding the presence and constant interference of two attendants, and the blows and bruises received in these saltations. The tendency to grovel is but a type of general degradation. Theory has suggested that many of these movements and practices, as well as the cries formerly described, are the imitation of the natural language and habits of the lower animals ; but it has been more philosophically advanced that the deranged mind being frequently reduced to an animal condition, finds expression in the same signs as those of the species to which it is most closely assimilated ; and, thirdly, that the part of the nervous system connected with the production of instinctive acts being the seat of morbid excitement, or its functions being no longer controlled by the will or judgment, prompts and produces these grimaces and grovellings, which are, upon ordinary grounds, inexplicable. It is probable, that viewed from another point, they may be traced to modifications of chorea ; but as emanating from exalted rather than from suspended volition. Where running and climbing are presented, the cause or prominent feature of the malady has generally been fear. We have seen climbers, even when exhausted by age and anxiety, although exposed to extreme danger, and actually hourly subjecting themselves to severe injury, clambering up walls and windows like athletes. In one the impulse was exquisite terror; he was compromised, condemned, lost: he fled from his own sensations. In another there predominated the dread of physical torture: his skin was on fire; his clothes blazed around him; he was about to be consigned to the flames; when his hair was cut he feared decapitation; when shaved that he was to share the fate of Cressingham. In place of attempting to ascend heights, insane persons sometimes walk or whirl in circles. They will turn invariably from right to left, or left to right, completing a certain and always the same number of circles, when addressed or required to move, or they may perform repeated somersets along the floor. Among the many indications of a diseased nervous system scattered through the history of Dr. Samuel Johnson, it is recorded that he always passed in or out of a door or passage by a certain number of steps, from some particular point, and invariably made his exit and his entrance with the same foot foremost. If he failed to do this correctly, he went back to his starting-place and began over again. Before he crossed a threshold he commonly turned round upon his heel and often stopped in the street to whirl in these magic circles.

In addition to the suffering which may arise from imaginary dangers, or from unconsciousness of their own infirmities, the insane are subjected to innumerable petty annoyances from irksome, or unpleasant, or actually painful impressions referred to the organs of sense. The uneasiness, the sensation, may be real; the error resides in the conclusion drawn as to its nature. They detect disagreeable sapors in every article of food. These may be attributed to poison, to the want of care or culinary art in preparing the food. There are others who, from similar but unacknowledged causes, do not swallow the saliva, but eject it laboriously and systematically. There are others tormented by noxious or oppressive odours which do not exist; or by sounds which arise in and are re-echoed within their own ears, or by shapes and visions which people the air around. Particular forms and colours inspire horror; occasionally certain colours are not perceived. According to Dr. George Wilson's inquiries this defect obtains largely among the healthy to the extent of 5·6 per cent. of those examined, and may be connate and hereditary. It is curious that these delusions, when occurring in delirium tremens, refer, in the great majority of cases, to objects of small size—to insects, seeds, cats, the pathognomic rat, and to devils which are not only blue but diminutive. A gentleman who was in the habit of inhaling ether, either experimentally or as a luxury, told us that for days subsequent to a debauch all objects around appeared to be of extremely small size and at a great distance, and although fully aware of the deception, and of its origin, he experienced some difficulty in adapting himself and regulating his movements in relation to the Lilliputian world of which he seemed to be an inhabitant. Peculiar sensations of softness, smoothness, hardness of the skin, neuralgia of the surface, the hallucination that the extremities are constantly galvanized, that the whole body is in a state of combustion, igniting the clothes and melting the iron framework of the bed, that it has increased enormously, or has diminished in size, appear to depend upon some altered condition of the sense of touch. Similar impressions are experienced during inebriety and under the influence of hachschich. They are the premonitors of paralysis in many cases. Saussure conceived that he had acquired such gigantic proportions that the doors were widened, the partitions removed for his accommodation, but in vain. A patient at one time under our care strenuously held that he was not merely reduced to the magnitude of a grain of corn, but that he was a grain of corn, and obstinately refused to go into the open air in case the sparrows should pick him up. But it is not only in acts or delusions that these minute deviations may be discovered, but in principles of action, habits of thought, in the exercise of feeling. A man has been haunted throughout life by a single meaningless monosyllable; a living but octogenarian senator is at all times conscious of the rustle and contact of bombazine; the whole inner life of one fanatic, his arrogation of the divine nature, his sublime incoherence, have revolved round his name "Dieu;" and a whole sect of enthusiasts, the Jumpers, who afford the most genuine example of muscular Christianity, conduct and defend their gyrations upon an interpretation of the text, "turn ye, turn ye, why will ye die?"

There is a fashion in the election of prominent symptoms as pathognomic in certain diseases, and in insanity as well as others. The colour of the skin and the pulse were formerly carefully noted: they are now disregarded, and all other considerations are in danger of being swallowed up in the search for delusions. It is natural that when these minor traits are solitary they may be passed over or undervalued, because a counterpart, or some analogous petty anomaly, may be the foible and foil of great faculties. When a poor lypemaniac puts his head into a hole, a parallel is found in Lord Bacon, who is reported to have pursued a similar practice curatively, and of having gone from London to Richmond Hill every morning to do it. We have had patients who fondled frogs and made pets of snails; but Lord Erskine felt gratitude to leeches, preserved them, introduced them to his friends, calling the one "Cline" and the other "Home," after the great surgeons of his day. The disposition to dissociate such acts and peculiarities from the mental conditions, and from the disease in which they originate, and to place them in a vague category, called automatic, and to ignore them as not bearing upon the issue of the case, have led to similar results. But these are not only susceptible of being referred to obvious principles and reduced to an intelligible system of classification, but they appear to be regulated by general laws. They are, for example, more frequent in chronic than in acute forms of alienation. They are the growth of the changed nature, the expression of the mutilation, or of the second childishness and mere oblivion which have been established. It may not be regarded as a microscopic object, that Robert Hall's imagination was dimmed, darkened; according to his own estimate, put out; by an attack of mania. Yet it was but the loss of a fraction of a mighty whole, and seems to have permitted the more solemn and stately exercise of the higher intellectual powers. In a patient of my own there was engrafted upon a disposition powerfully shaken by the mania of suspicion, the habit of incessant expectoration. These slight peculiarities are the offspring of certain forms of mental affection rather than of others; and dementia is most prolific. They may be modified, diverted, trained, eradicated. The actors, artists, authors, the butts, buffoons, the celebrities, even the industrious in asylums, often owe their position to a petty fantasy, or to the direction and development of such a motive by those around, and not to the ordinary motives and objects which create such classes in the world. We have seen two Simon Stylites stand for hours as statues in a tableau, and serve as candlesticks in an entertainment. Lee wrote some of his poetry, not merely while he ordered Jupiter to snuff the moon, but because he conceived that Jupiter obeyed. We have known a man toil for a twelvemonth in order to raise a crop of tea; and the quaintness and witticisms of the dement, although they cannot be traced, as in the case of Motley, to his dress, may fairly be so to the hap-hazard of disjointed incoherence.

Further, they do not necessarily cease with the malady in which they originated. Whether recognised as a part of a disease which has been arrested or remained undeveloped, or as one of a group of symptoms, or as a disease or group of symptoms in themselves, in the same way that instances of local epilepsy, or the convulsion of a single muscle, as in the zygomatici of a retired Lord Chancellor, are met with, they justify an unfavourable prognosis, and the suspicion that the nervous centres are either positively diseased, or retain that susceptibility to unhealthy action which is so often observed subsequently to mania. The patient whose habit of spitting has been alluded to, committed suicide after an apparently lucid interval of twenty years' duration.

There is, besides, a broad and practical view of this matter. It is a daily observation that individuals are so slightly insane, afford such indefinable or inappreciable evidence of their difference from other and healthy men, that it strains ingenuity and taxes conscience to deprive them of supposed rights. This dilemma may arise from the actual absence of the familiar and unequivocal features of disease, or from the dexterity of the patient in withdrawing the outward and visible indications of his condition, and in affecting intelligence and prudence which he does not possess. He may understand the symptoms of alienation, he may be a better actor than his inquisitor. Experts are baffled, not so much in forming an opinion, as in discovering and assigning, or in presenting with clear and sufficiently large proportions, the facts upon which that judgment is founded. Their inference may be founded upon a look, a gait, a piece of dress, or, in the phraseology of a certificate submitted to a Board of Lunacy, upon the fact that the patient "spoke fast, and wore a white hat;" or upon some faint, or delicate, or ephemeral deviation from a standard created by the observer—a deviation which may not be perceived by others, or may be perceived in those of robust and unwarped judgment. The data may, however, be most significant, they may often be the salient point of a deep and extensive disorganisation. The most appalling murders have been committed by maniacs who, superficially viewed, possessed a reputation for sanity. The importance of a sign must not be estimated by the space which it occupies in the field of observation, or by the number or value of the functions which it involves. A dilated pupil, or a dream, or an attack of convulsions, may be of equal value as a guide to prognosis. It is the characteristic of a penetrating and comprehensive mind to detect in faint foreshadowings great and distant results; and in some instances the difficulty in defining the point at which the limits of reason have been passed, arises from the inability of the observer to connect the sign with the mental change to which it corresponds, or from his inability to convince others of this connexion. It is, besides, the element of many deep-seated species of mental maladies, to give no sign, or nothing but faint and uncertain signs. Were a diagnosis founded upon the conviction entertained by an elderly clergyman that he could walk eight miles in an hour, it would be laughed at; yet in that conviction there is contained, not potentially, but the actual germ, the morbid element of preternatural strength or swiftness, the first glimmering of the optimism of general paralysis. There is a fatal obtuseness in overlooking mere exaggerations of the ordinary qualities of mind because they are trivial and harmless.

There was sagacity as well as sarcasm in the observation, that the use of language was to conceal thought. There is a secret skeleton in every heart. The greatest triumphs of mind, and cunning and art, have perhaps consisted in shrouding this spectre deformity from the public eye. There is self-deception in the effort, and the victim persuades himself that he is emancipated from his bonds because he has succeeded in simulating freedom. It has sometimes been a sustained life-struggle to cast far back from observation or suspicion into the darkest depths of the heart, the perverted purpose, the solitary morbid association, the simulacrum of madness. And the amount of aberration successfully concealed is sometimes astounding. The decollated head which glared upon the late Lord Grey, one of our most serene and self-possessed statesmen, while he was in the senate or at his own fireside, during the animation and excitement of party debate, and during the calm of solitary reflection, was a personal sorrow and suffering, and not confessed to his family, or to his family only. But the pain, and the solicitude, and the ingenuity in avoiding disclosure and defeating inquiry, are not always in proportion to the size of the plague-spot. In that confessional, the consulting-room, a lady lately disclosed to us that she hears at all times, and under all and the most incongruous circumstances, not merely sounds in her ears and head, but tunes which she can generally distinguish, to the measure of which she is strongly disposed to move her head, but dreads the detection which would attend indulgence in this impulse. There is reason to believe that to some mental or moral obliquity, so subtle or so slight as to escape or baffle observation, may be attributed some of the instances of eccentricity and extravagance which disturb or disorganize every community, where, although the source be latent or remote, there is no desire to withdraw the manifestation from publicity, and no suspicion that it is regarded as absurd. That a man of science should open his window precisely at two o'clock every morning, and blow his nose audibly and ostentatiously for an hour, appears, at first, simply ridiculous; but inquiry shows that the practice may have originated in the periodicity by which his actions were greatly guided, in erroneous conceptions as to health, and that it was the solitary revelation of a morbid nervous system.

